# Small ankyrin 1 (sANK1) promotes docetaxel resistance in castration‐resistant prostate cancer cells by enhancing oxidative phosphorylation

**DOI:** 10.1002/2211-5463.13535

**Published:** 2022-12-26

**Authors:** Yang Yang, Haixiang Qin, Meng Ding, Changwei Ji, Wei Chen, Wenli Diao, Haoli Yin, Mengxia Chen, Weidong Gan, Hongqian Guo

**Affiliations:** ^1^ Department of Urology, Drum Tower Hospital, Medical School of Nanjing University, Institute of Urology Nanjing University China

**Keywords:** docetaxel resistance, OXPHOS, prostate cancer, PTBP1, sANK1

## Abstract

Docetaxel (DTX) plays an important role in treating advanced prostate cancer (PCa). However, nearly all patients receiving DTX therapy ultimately progress to DTX resistance. How to address DTX resistance in PCa remains a key challenge for all urologists. Small ankyrin 1 (sAnk1) is an integral membrane protein in the endoplasmic reticulum. In the present study, we identified that sAnk1 is upregulated in PCa tissues and is positively associated with DTX therapy resistance in PCa. Further investigation demonstrated that overexpression of sAnk1 can significantly increase the DTX‐resistant ability of PCa cells *in vitro* and *in vivo*. In addition, overexpression of sAnk1 could enhance oxidative phosphorylation (OXPHOS) levels in PCa cells, which was consistent with the higher OXPHOS levels observed in DTX‐resistant PCa cells as compared to DTX‐sensitive PCa cells. sAnk1 was also found to interact with polypyrimidine‐tract‐binding protein (PTBP1), an alternative splicing factor, and suppressed PTBP1‐mediated alternative splicing of the pyruvate kinase gene (*PKM*). Thus, overexpression of sAnk1 decreased the ratio of PKM2/PKM1, enhanced the OXPHOS level, and ultimately promoted the resistance of PCa cells to DTX. In summary, our data suggest that sAnk1 enhances DTX resistance in PCa cells.

## Graphical abstract

sANK1 is localized to the sarcoplasmic reticulum/endoplasmic reticulum, and it might bind to PTBP1 and restrain its entry into the nucleus to mediate alternative slicing of PKM. Overexpression of sANK1 increases the level of PKM1 and enhances OXPHOS in prostate cancer by suppressing the function of PTBP1. Enhanced OXPHOS promotes resistance of prostate cancer cells to DTX treatment.

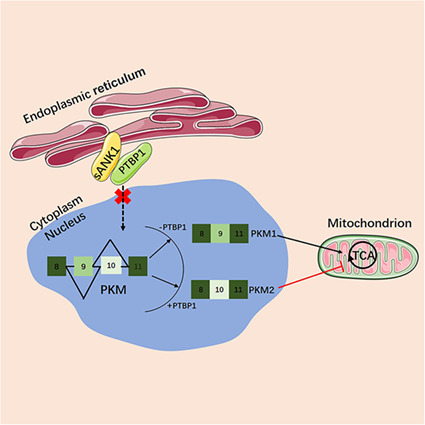

**Abbreviations**
ADT: androgen‐deprivation therapy
BSA: bovine serum albumin
CRPC: castration‐resistant PCa
DR: DTX resistant
DTX: docetaxel
IHC: immunohistochemistry
OCR: oxygen consumption rate
OXPHOS: oxidative phosphorylation
PK: pyruvate kinase
sANK1: small ankyrin 1
SR/ER: reticulum/endoplasmic reticulum
TMA: tissue microarray
WB: western blot

Metastasis is still a lethal factor of prostate cancer (PCa), with the second highest mortality of cancers in men in developed countries [1]. In recent years, the incidence of PCa in China has strikingly increased [2]. Patients diagnosed with early‐stage PCa have a good prognosis, with a 5‐year survival of nearly 100%, whereas only 30% of patients with metastatic disease can achieve a 5‐year survival [3]. Despite the initial effectiveness of androgen‐deprivation therapy (ADT) in treating advanced and metastatic PCa, nearly all patients finally progress to castration‐resistant PCa (CRPC) [4]. As the first line of defense, chemotherapy is the treatment for CRPC, which means patients could benefit from docetaxel (DTX) treatment. However, almost all patients accepting DTX treatment ultimately become refractory because of DTX resistance [5]. Therefore, investigating the molecular mechanisms underlying DTX resistance has great clinical significance with potential for novel strategies to treat DTX‐resistant PCa.

To date, many studies have focused on the molecular mechanisms of DTX resistance in advanced PCa. Abnormal overexpression of multidrug resistance genes in tumor cells represents one of the most extensively investigated mechanisms of chemotherapy resistance [6, 7]. Multidrug resistance genes, such as ABCB1 and ABCC4, have been reported as upregulated and may contribute to DTX resistance in PCa [7, 8]. In addition, some studies indicate that β‐tubulin isotypes may affect the response of cancer cells to microtubule‐targeting drugs [9]. For example, βIII‐tubulin has been reported to be elevated in DTX‐resistant cells and showed an association with the response of PCa to DTX‐based chemotherapy [10]. Although enormous progress has been achieved in the study of docetaxel‐resistant PCa, few agents can be used in current clinical settings due to severe or poor side effects [11]. Recent studies suggest that metabolomic changes unique to drug‐resistant cancer cells may hold the key to reversing drug resistance in cancer [12]; however, numerous studies are required to probe the metabolic characteristics and reprogramming mechanisms of DTX‐resistant PCa cells [12].

As an integral membrane protein of the ankyrin family, ankyrin 1 (ANK1) functions as a protein adaptor in the organization of specialized membrane domains [13]. In recent studies, ANK1 has been reported to regulate glucose uptake in skeletal muscle, and alteration of the expression of ANK1 may induce insulin resistance [14, 15]. The small ankyrin 1 (sANK1), a small (~ 17 kDa) ankyrin isoform, is a short alternative splicing of the *Ank1* gene [16]. Our previous study found that miR‐486‐5p, which shared the same promotor with sANK1 [17], was upregulated and suppressed multiple tumor suppressor pathways in PCa, playing a critical role in PCa progression [18]. Moreover, we found that sANK1 was obviously upregulated in PCa tissues compared to adjacent normal tissues and was also upregulated in the PCa tissues of DTX‐resistant patients rather than in DTX‐sensitive patients in the present study. Moreover, overexpression of sANK1 can significantly increase DTX resistance and simultaneously enhance oxidative phosphorylation (OXPHOS) levels in PCa cells. Therefore, the focus of this study is on the function and mechanism of sANK1 during the progression of DTX‐resistant PCa.

## Materials and methods

### Tissue samples and tissue microarray

The Ethics Committee of Drum Tower Hospital, Medical School of Nanjing University, approved this study, which was consistent with the Declaration of Helsinki principles (approval code: 2018‐165‐01). Written informed consent has been obtained from each patient. Formalin‐fixated prostate cancer tissues (*N* = 36) for immunohistochemistry as well as frozen prostate cancer and adjacent normal tissues for quantitative real‐time PCR (*N* = 20) were collected from patients undergoing prostatectomy from 2019 to 2020. All these patients underwent a prostatectomy prior to the receipt of any adjunctive therapy. The frozen tissues have been made into frozen sections to confirm the histopathological features by an experienced pathologist. The DTX‐sensitive tissues were obtained from patients undergoing the first biopsy before DTX treatment, and the corresponding DTX‐resistant tissues were obtained from the same patients undergoing another biopsy after they were resistant to DTX chemotherapy (*n* = 4). DTX chemoresistance was defined as patients receiving ADT and docetaxel chemotherapy whose serum testosterone concentration had reached a castration level which is less than 50 ng·dL^−1^ or 1.7 nmol·L^−1^ with any of the following progression: (a) biochemical progression: the consecutive PSA increases three times, in which the interval was more than 1 week and two of the three progressions were 50% higher than the nadir PSA level with a PSA level of more than 2 ng·mL^−1^. (b) Radiographic progression: more than two new bone metastases on bone scans or appearance of an enlarged soft tissue on the scans were evaluated through Response Evaluation Criteria in Solid Tumors (RECIST) criteria. The clinical characteristics of enrolled patients are listed in Tables 1 and 2.

**Table 1:** Characteristics of patients whose samples were used for IHC (*n* = 36) and qPCR (*n* = 20). IQR, interquartile range; ISUP, International Society of Urological Pathology; PSA, prostate‐specific antigen.

Characteristics	IHC (*n* = 36)	qPCR (*n* = 20)
Age (years), median (IQR)	67 (62–73)	69 (64–72)
Initial PSA (ng·mL^−1^), median (IQR)	9.24 (4.82–27.79)	7.81 (5.03–25.75)
ISUP grade, *n* (%)		
2	11 (30.6)	6 (30)
3	16 (44.4)	9 (45)
4	5 (13.9)	3 (15)
5	4 (11.1)	2 (10)
Pathological T stage, *n* (%)		
pT1/pT2	29 (80.6)	15 (75)
pT3/pT4	7 (19.4)	5 (25)

**Table 2:** Characteristics of four patients who were resistant to DTX chemotherapy. ISUP, International Society of Urological Pathology; PFS, progression‐free survival; PSA, prostate‐specific antigen.

Patient	Age	Initial PSA (ng·mL^−1^)	Nadir PSA (ng·mL^−1^)	cTNM	ISUP grade	PFS (month)
Case1	70	478	0.796	T4N1M1b	5	6
Case2	78	2719	1.832	T3bN1M1b	4	11
Case3	73	642	0.887	T3bN1M1b	5	5
Case4	65	3224	20.73	T3bN1M1b	5	4

A tissue microarray (TMA) was constructed with one tissue core (2 mm in diameter) from a representative area of each sample identified by two experienced pathologists. A signed consent was obtained from each patient.

### Cell lines and cell culture

Human PCa cell lines (Du145 and PC‐3) were purchased from the National Collection of Authenticated Cell Culture (Shanghai). Cells were cultured in RPMI 1640 medium with 10% fetal bovine serum (FBS), 100 U·mL^−1^ penicillin, and 100 mg·mL^−1^ streptomycin. All the cells were placed in a moist environment with 5% CO_2_ at 37 °C.

### DTX‐resistant cell generation

Du145 and PC‐3 cells were cultured in a medium consisting of 5 nm DTX for 24 h. The cultured cell medium was then replaced with normal RMPI 1640 medium. When the cells continued to proliferate, 5 nm DTX was added. While the cells could proliferate in the medium with 5 nm DTX, they were treated with a higher concentration of DTX. DTX‐resistant cells were defined as DTX‐treated cells with an inhibitory concentration of 50% (IC_50_), 10 times higher than that of the parental cells.

### Immunohistochemistry (IHC) and immunofluorescence (IF)

Tissue microarray and the biopsy tissue specimen were used for IHC. Immunostaining was assessed independently by a pathologist in a blinded manner. The staining intensity was scored as 0 (negative), 1 (weak), 2 (intermediary) and 3 (strong), while the staining range was scored as 0 (0%), 1 (1–25%), 2 (26–50%), 3 (51–75%) and 4 (75–100%), which combined and resulted in scores on a scale of 0–12. The staining intensity of the biopsy tissue was calculated by image‐pro plus 6.0 (Media Cybernetics, Inc., Bethesda, MD, USA).

Immunofluorescence (IF) was performed to detect the protein distribution in cells. Cells were cultured in a 48‐well plate and fixed with 4% paraformaldehyde (PFA) (w/v). The cells were permeabilized in 0.3% Triton X‐100 (v/v) (Sunshine Biotech, Nanjing, China) diluted in PBS, then 3% (w/v) bovine serum albumin (BSA) (Sangon Biotech, Shanghai, China) was used to block the cells for 1 h. The primary antibody was then added to the wells, and the plate was placed in a 4 °C environment overnight. The cells were incubated with fluorescence‐labeled secondary antibody (CST, Danvers, Massachusetts, USA) for 1 h at room temperature and finally treated with Sigma 4′,6‐Diamidine‐2′‐phenylindole dihydrochloride (DAPI) for 2 min. All incubations were followed by three PBS washes.

### Quantitative real‐time PCR (qRT‐PCR) analysis

qRT‐PCR was performed as described previously [18]. Briefly, the total RNA was extracted from adjacent normal tissue and prostate cancer by TRIzol reagent and reverse transcribed to cDNA using PrimeScript RT Master Mix (TaKaRa Biotech, Nojihigashi，Japan). Meanwhile, SYBR Master Mix (Vazyme, Nanjing, China) was used for qRT–PCR on the QuantStudio^‐^™ 6 Flex System (PE Applied Biosystems, Foster City, CA). The relative expression of mRNA was normalized to ACTB by the $2^{-\Delta C_{t}\Delta C_{t}}$ method. The forward primer sequence of sANK1 was F 5′GGAGACCATCTCCACCAGG 3′, and the reverse primer sequence was R 5′ CCACCTTGCGAATGATCTTCT 3′. The forward primer sequence of ACTB was F 5′ CATGTACGTTGCTATCCAGGC3′, and the reverse primer sequence was R 5′ CTCCTTAATGTCACGCACGAT 3′.

### Western blot analysis

Western blot (WB) analysis was performed as described previously [19]. Briefly, RIPA buffer containing protease inhibitor and phosphatase inhibitor was used to solubilize the cells, and the lysate was centrifuged at 16,000 *g* for 20 min. The supernatant was collected and boiled at 95 °C with loading buffer for 5 min and used for WB analysis. Tubulin was selected as the reference gene. Antibodies against sANK1 and PKM2 were purchased from Abcam (Cambridge, UK), and antibodies against PTBP1 and tubulin were purchased from Proteintech Group (Wuhan, China).

### Plasmid construction, lentiviral infection and oligonucleotide transfection

The coding sequence of human sANK1 was amplified by PCR with the forward primer F: 5′GACGATGACAAGCTTGCGGCCGCTATGTGGACTTTCGTCACCCA‐G3′ and the reverse primer R: 5′GATCGCAGATCCTTCGCGGCCGCTCACTGTTT‐CCCCCTTTTCAG3′ and inserted into the multiple cloning site of the lentiviral vector pCDH‐CMV‐3 × FLAG‐ZHX3‐EF1‐puro (pCDH). Then, the vector was cotransfected into HEK293T cells along with a three‐plasmid expression system. Forty‐eight hours after transfection, a 0.2 μm filter was used to filter the supernatant, which was then collected and stored at −80 °C. Du145 and PC‐3 cells were infected with the lentivirus supernatant and screened with puromycin at 48 h after infection. SiRNA or negative control (siNC) was obtained from GeneChem (Shanghai, China) and transfected into Du145‐DR and PC‐3‐DR cells with Lipo2000. The overexpressed plasmid of PKM2 was purchased from YouBio (Changsha, China).

### MTT assay

The IC_50_ of DTX in PCa cells by MTT assay. Briefly, 5000 cells were seeded into 96‐well plates. When cells were adherent, medium with DTX in different concentration ranges (1 nm to 256 nm) was added to the wells. After 48 h, 10 μL MTT solution was added to the wells. After 2 h, the solution was removed. To dissolve the purple formazan crystals, DMSO (100 μL) was added to the wells, and the plates were placed in a 37 °C environment for 30 min. Absorbance was measured at 490 nm, while 630 nm was selected as a reference using a microplate reader (Tecan Infinite, Männedorf, Switzerland).

### Mass spectrometry

Cell lysates of Du145‐sANK1 and PC‐3‐sANK1 cells were immunoprecipitated with anti‐FLAG antibody. The mass spectrometry data acquisition and analysis were completed at GeneChem. In brief, the protein solution was digested by protease into a mixture of peptides, which were subjected to NSI source followed by tandem mass spectrometry (MS/MS) in Q ExactiveTM Plus (ThermoFisher Scientific, Waltham, Massachusetts, USA) coupled online to the UPLC. The resulting MS/MS data were processed using proteome discoverer 1.3 (ThermoFisher Scientific).

### Co‐immunoprecipitation (Co‐IP)

Cell lysates of Du145‐sANK1 and PC‐3‐sANK1 cells were incubated with protein A/G agarose beads one time and then incubated with an anti‐FLAG antibody and control IgG overnight with the protein A/G agarose beads. The complexes were washed three times with lysis buffer and resuspended in 2× SDS loading buffer. The immunoprecipitated proteins were eluted from the beads by incubation at 95 °C for 5 min. The eluted proteins were detected by WB.

### Measurement of intracellular calcium

Cells were digested by tyrosine without EDTA and washed with PBS two times. Then, the cells were resuspended and loaded with Fluo 3‐AM (5 μm) for 30 min at 37 °C. The loaded cells were rinsed three times with PBS and then intracellular Ca^2+^ was determined by a flow cytometer (BD Sciences, Franklin Lakes, New Jersey, USA) at an excitation wavelength of 488 nm and an emission wavelength of 525–530 nm.

### Glucose uptake and deprivation assay

Glucose uptake and deprivation assays were performed by a flow cytometer (BD Sciences). Glucose uptake was assessed by 2‐NBDG, a fluorescent glucose analog. Cells were cultured in 12‐well plates and treated with 2‐NBDG for 2 h after 2 h of glucose deprivation. Then, flow cytometry was used to analyze the 2‐NBDG uptake at excitation and emission wavelengths of 465 and 540 nm, respectively. For the glucose deprivation assay, cells were seeded into 24‐well plates, and the medium was replaced with glucose‐free medium (Gibco, ThermoFisher Scientific) for 12 h. Apoptosis was measured by flow cytometry with an Annexin V‐FITC Apoptosis Detection Kit (Vazyme).

### Metabolic assay

A Seahorse XF96 analyzer (Seahorse Biosciences, Billerica, Massachusetts, USA) was used to assess the oxygen consumption rate (OCR), which reflects the rate of OXPHOS. A total of 15 000 cells were seeded into a 96‐well XF96 plate and cultured overnight. The Cell Mito Stress Test Kit (Agilent Technologies Inc., Santa Clara, California, USA) was used to measure cellular mitochondrial flux. Several inhibitors, oligomycin (1.25 μm, mitochondrial ATP synthase inhibitor), carbonyl cyanide 4‐(trifluoromethoxy) phenylhydrazone (FCCP) (2.5 μm, protonophore and uncoupler of ATP synthesis from mitochondrial respiration), and rotenone (0.75 μm, electron transport inhibitor) were added according to the manufacturer's instructions. ATP production and spare respiratory capacity were two important indicators to reflect the ability of OXPHOS. At basal respiration, OCR decreased significantly after oligomycin was added to inhibit ATPase; reducing OCR was represented to the ability of ATP production. After adding the FCCP uncoupling agent, electron transport would lose the constraint of proton gradient and move at the maximum speed, enabling OCR to increase sharply to the maximal oxygen consumption. At this time, the difference between the maximal oxygen consumption and basic respiration is called spare respiratory capacity.

### Animal study

Six‐week‐old male nude mice were purchased from the Model Animal Research Center of Nanjing University. A total of 5 × 10^6^ cells in each group were subcutaneously injected into the two flanks of mice (left: Du145‐NC, right: Du145‐sANK1, *n* = 6). The large (*L*) and small (*S*) diameters of the tumors and the weight of the mice were measured every 4 days, and the tumor volume was calculated using the formula *S* ^2^ × *L*/2. Four weeks later, these mice were treated with DTX (5 mg·kg^−1^) once a week for 3 weeks continuously. One week after the final treatment, the mice were sacrificed by euthanasia, and the tumor tissues were harvested. All animal experiments were performed according to the guidelines of the Ethics Committee of Animal Research of Nanjing Drum Tower Hospital.

### Statistical analysis

The data analysis was performed with graphpad prism 6 (San Diego, California, USA) and ibm spss statistics 17.0 (International Business Machines Corporation, Armonk, New York). The repeated measurement data were analyzed by the chi‐square test. Normally distributed data are expressed as the mean ± SD and were compared by Student's *t*‐tests. *P* < 0.05 was considered statistically significant.

## Results

### sANK1 was upregulated in chemoresistant PCa

**Fig. 1:**
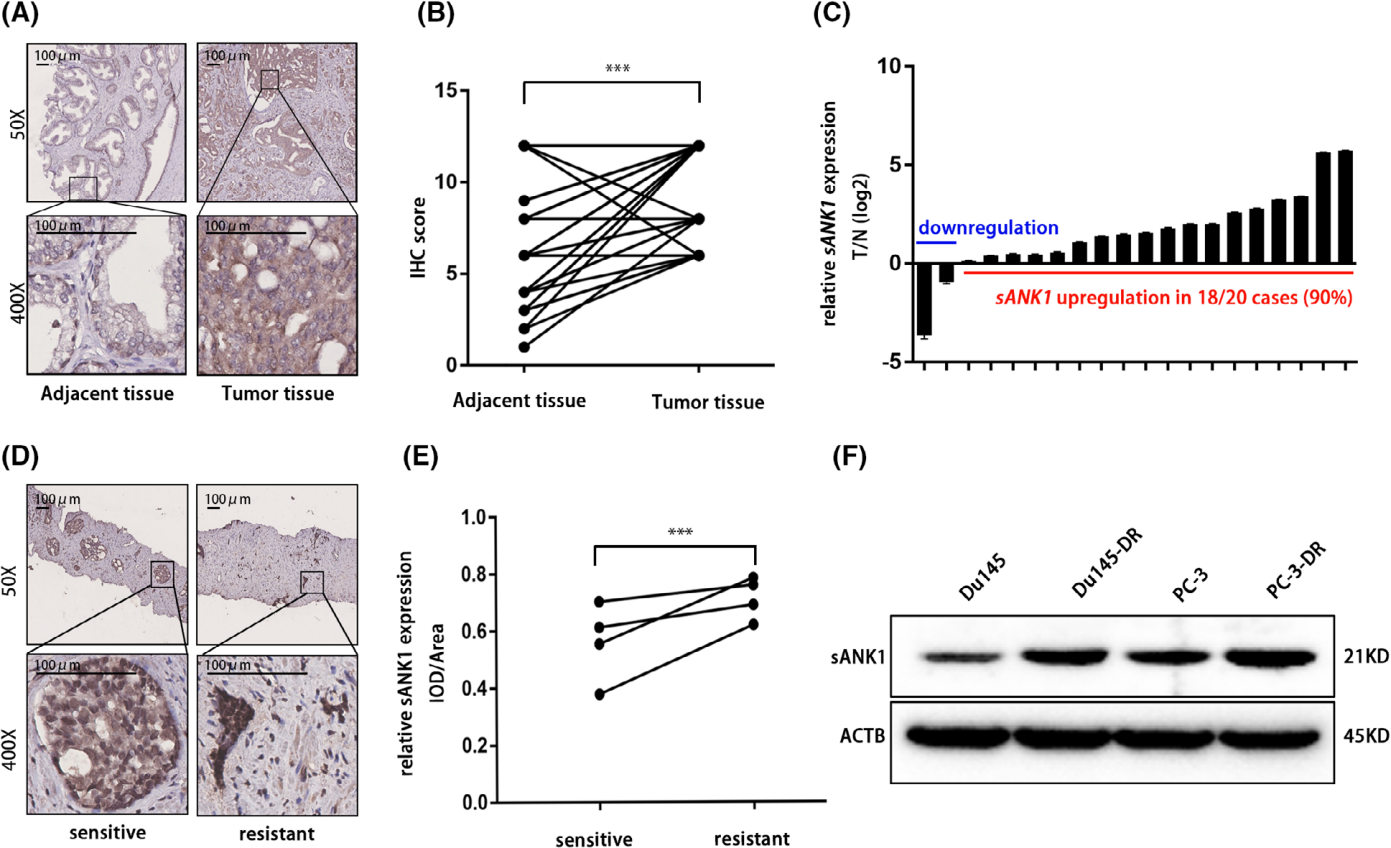
sANK1 was overexpressed in PCa, and its expression was higher in DTX‐resistant PCa. (A, B) IHC analysis shows that the expression of sANK1 in PCa tissue was higher than that in the adjacent tissue. Scale bar, 100 μm. *n* = 36. *P* values, by Student's *t‐*test. ***P < 0.001. (C) qPCR results show that sANK1 was overexpressed in PCa tissue at the mRNA level. *n* = 20. *P* values, by Student's *t*‐test. (D, E) Biopsy samples of patients who were resistant to DTX treatment were tested by IHC, and the results implied that the expression of sANK1 was higher in DTX‐resistant PCa. Scale bar, 100 μm. *n* = 4. *P* values, by paired‐samples *t*‐test. ***P < 0.001. (F) sANK1 was overexpressed in DTX‐resistant cell lines compared to their parental cells.

We first investigated the sANK1 protein levels in PCa tissues and paired adjacent normal tissues by IHC staining. The IHC results revealed that PCa tissue had a higher sANK1 level than the adjacent normal tissue (Fig. 1A,B). We also analyzed the mRNA expression of sANK1 in 20 pairs of PCa and adjacent tissues. Higher sANK1 mRNA expression was observed in 18 of 20 cases (Fig. 1C). Then, we compared the sANK1 protein levels in chemoresistant PCa tissues with those in chemosensitive PCa tissues and found that sANK1 was upregulated in chemoresistant PCa tissues (Fig. 1D,E). Next, we constructed two chemoresistant PCa cell lines (PC‐3‐DR and Du145‐DR) and further investigated sANK1 expression in those cell lines. As shown in Fig. 1F, chemoresistant cell lines had higher sANK1 expression level than their parental cells. These results indicated that compared to normal prostate tissue, sANK1 was overexpressed in PCa tissues and showed a positive correlation with the chemotherapy resistance of PCa.

### Changing chemosensitivity in PCa through dysregulation of sANK1

**Fig. 2:**
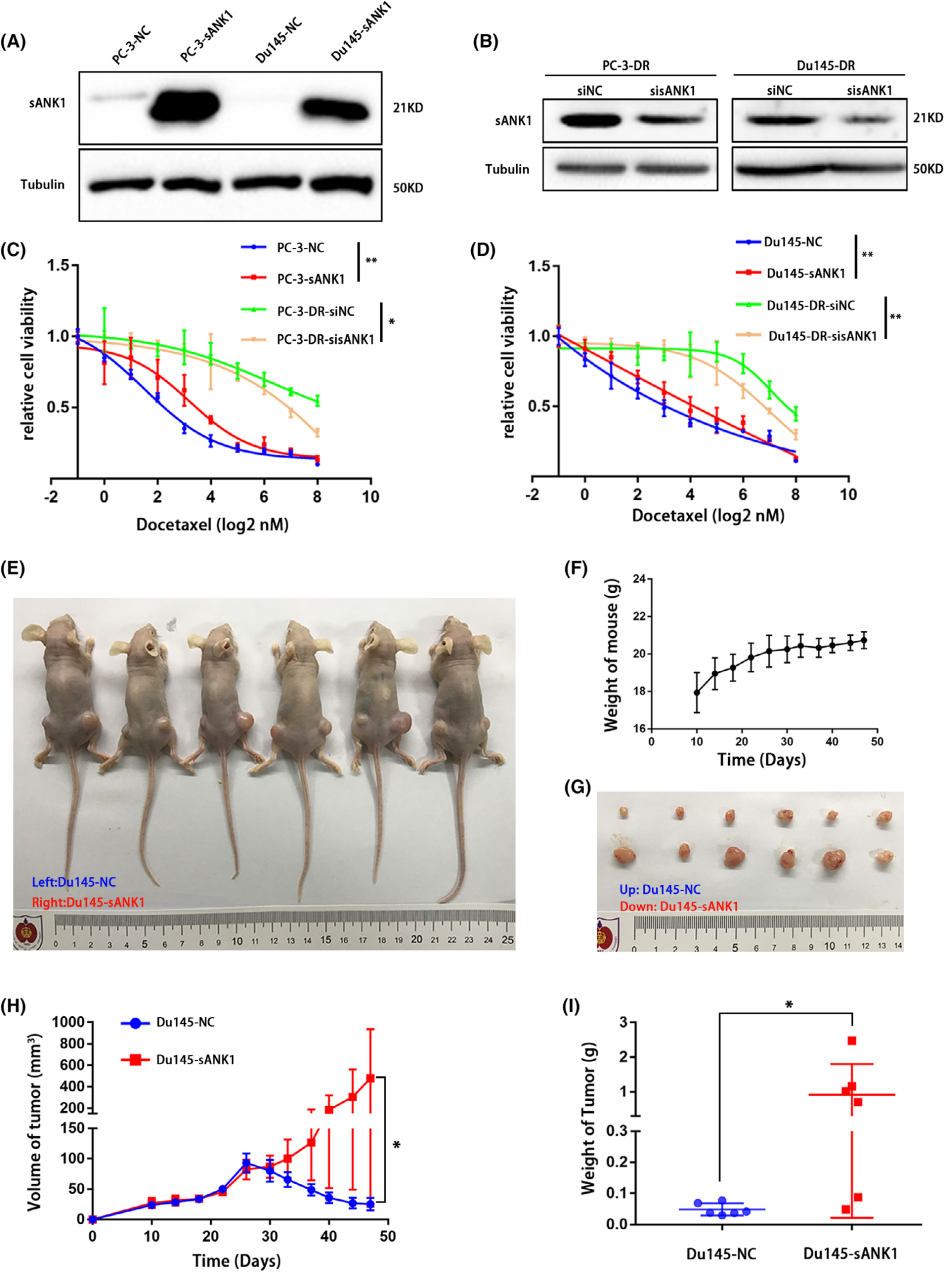
sANK1 enhanced resistance to docetaxel in prostate cancer. (A, B) sANK1 was overexpressed in PC‐3 and Du145 cells, although lentivirus and knocked down in PC‐3‐DR and Du145‐DR cells by siRNA. (C, D) Overexpression of sANK1 induced DTX resistance in both PC‐3 and Du145 cells, while knockdown of sANK1 resensitized PC‐3‐DR and Du145 cells to DTX. *n* = 3. *P* values, by chi‐square test. *P < 0.05, **P < 0.01. Data are mean ± SD. (E–I) Comparison of tumor engraftment size and weight in nude mice injected with Du145‐NC and Du145‐sANK1 cells with a 3‐week treatment of DTX. During the DTX treatment period, the weight of nude mice did not decrease. *n* = 6. *P* values, by Student's *t*‐test. *P < 0.05. Data are mean ± SD.

Considering the overexpression of sANK1 in chemoresistant PCa tissues and cells, we hypothesized that sANK1 might play more than one role in PCa DTX resistance. To confirm this hypothesis, we constructed sANK1‐overexpressing cell lines (Du145‐sANK1 and PC‐3‐sANK1) with a sANK1‐overexpressing lentivirus and knocked down the sANK1 level in DTX‐resistant cell lines (Du145‐DR and PC‐3‐DR) using specific siRNA for sANK1. WB results confirmed the efficiency of overexpression and knockdown of sANK1 (Fig. 2A,B). MTT assays were performed to evaluate the impact of the altered sANK1 expression on DTX sensitivity in PCa cells. Cells with sANK1 overexpression showed more resistance to DTX treatment than the control group cells, and knocking down sANK1 in Du145‐DR and PC‐3‐DR cells obviously increased the cell sensitivity to DTX (Fig. 2C,D). Considering the effect of sANK1 in PCa *in vitro*, we further investigated whether the dysregulation of sANK1 affected the sensitivity of PCa to DTX *in vivo*. Two flanks of nude mice were subcutaneously injected with Du145‐NC and Du145‐sANK1 cells. After 4 weeks, these mice were treated with DTX intraperitoneally once a week and three times continuously. Compared to that in the Du145‐NC group, both tumor volumes (Fig. 2E,G,H) and tumor weight (Fig. 2I) in the Du145‐sANK1 group were significantly augmented after a 3‐week treatment with DTX. Ten days after implantation, the weight of the mice was measured every 4 days, and the mice showed no obvious weight loss, suggesting that the DTX dose was appropriate (Fig. 2F). The above results demonstrated that sANK1 could significantly enhance the DTX resistance of PCa cells *in vitro* and *in vivo*.

### Overexpression of sANK1‐induced chemoresistance by restraining the polypyrimidine‐tract‐binding protein 1 (PTBP1) localization

**Fig. 3:**
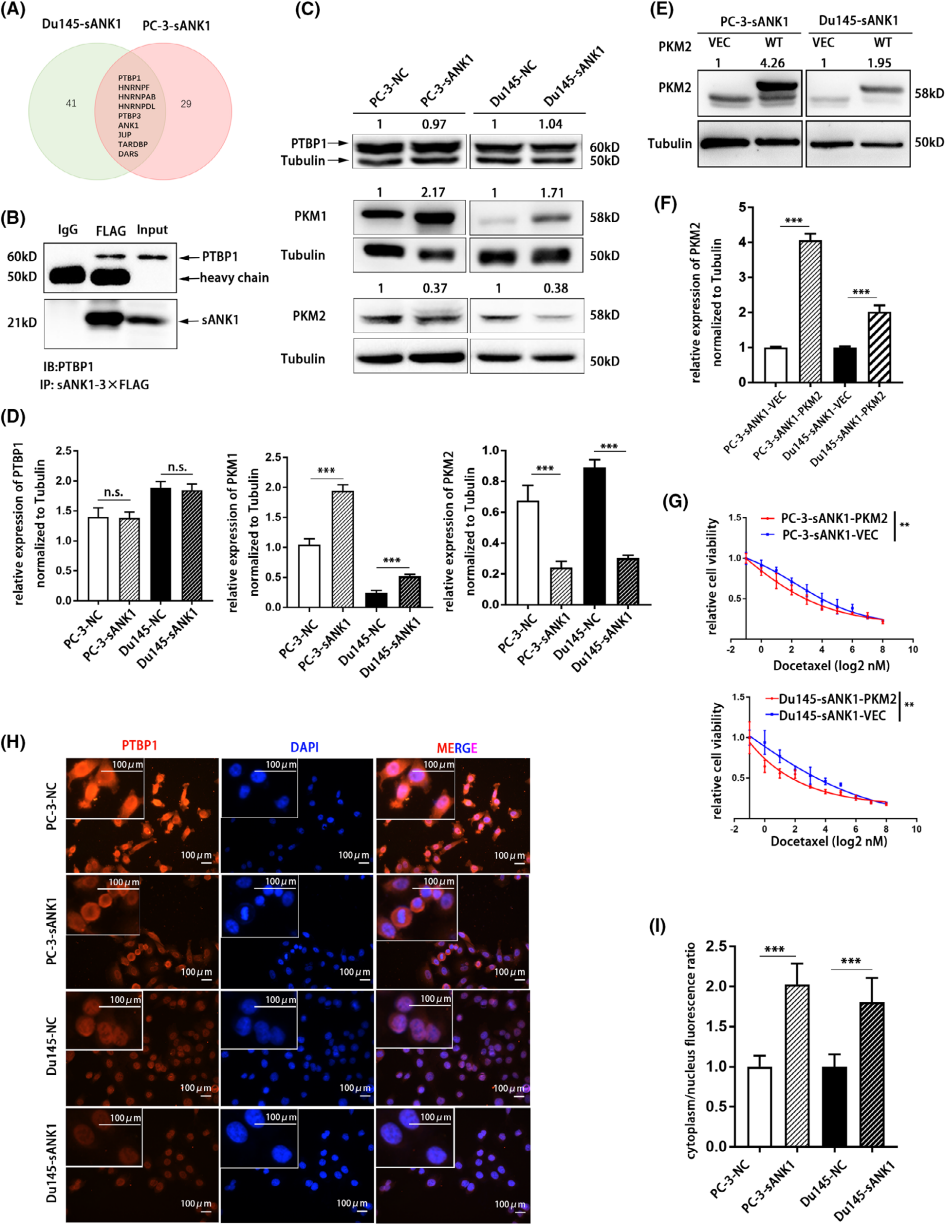
Overexpression of sANK1‐induced chemoresistance by restraining PTBP1 localization. (A) Venn diagram of mass spectrometry performed in PC‐3‐sANK1 and Du145‐sANK1 cells using IgG and FLAG antibodies. (B) WB analysis of interactions between PTBP1 and sANK1. (C) WB analysis of protein levels in PCa cells overexpressing sANK1. (D) Histogram statistics of three independent assays of C. *n* = 3. *P* values, by Student's *t*‐test. n.s., not significant, ***P < 0.001. Data are mean ± SD. (E) WB analysis of protein levels in PCa cells overexpressing PKM2. (F) Histogram statistics of three independent assays of E. *n* = 3. *P* values, by Student's *t*‐test. ***P < 0.001. Data are mean ± SD. (G) Overexpression of PKM2 resensitized Du145‐sANK1 and PC‐3‐sANK1 cells to DTX treatment. *n* = 3. *P* values, by chi‐square test. **P < 0.01. Data are mean ± SD. (H) Representative images from immunofluorescence with PTBP1 antibody in PCa‐overexpressing sANK1. Scale bar, 100 μm. (I) Histogram statistics of the cytoplasm/nucleus fluorescence ratio of PTBP1 in H. *n* = 3. *P* values, by Student's *t*‐test. ***P < 0.001. Data are mean ± SD.

To further reveal the mechanism by which sANK1 promoted the DTX resistance ability of PCa cells, we proceeded to identify the downstream target of sANK1 in DTX‐resistant PCa cells based on the location of sANK1 on the sarcoplasmic reticulum/endoplasmic reticulum (SR/ER) as a membrane protein. It has also been reported that sANK1 can interact with sarco‐(endo)plasmic reticulum Ca^2+^‐ATPase (SERCA1) [20], which led to the hypothesis that sANK1 mediates DTX resistance in PCa via protein–protein interactions. Therefore, we performed a Co‐IP assay and analyzed the potential target proteins by mass spectrometry. The mass spectrometry and subsequent WB results indicated that sANK1 could interact with PTBP1 (Fig. 3A,B). PTBP1 was reported to mediate the alternative splicing of PKM mRNA to generate two different isoforms (PKM1 and PKM2) [21]. We thereby detected alterations in the expression of PKM2 and PKM1 in PC‐3‐sANK1 and Du145‐sANK1 cells. The results showed that overexpression of ANK1 could increase the slicing of the PKM1 isoform and inhibit the slicing of the PKM2 isoform without affecting the protein level of PTBP1 (Fig. 3C,D). Furthermore, we restored PKM2 expression in Du145‐sANK1 and PC‐3‐sANK1 cells (Fig. 3E,F), and found their sensitivity to DTX was obviously enhanced (Fig. 3G). These results suggested that sANK1 induced DTX resistance through PTBP1‐PKM2 signal. Considering that sANK1 is a membrane protein in the ER, we hypothesized that sANK1 might bind to PTBP1 and restrain its entry into the nucleus to mediate alternative slicing. The immunofluorescence showed that PTBP1 distributed in the cytoplasm in sANK1‐overexpressing cells increased compared with that in control cells, and PTBP1 distributed throughout the nucleus correspondingly decreased (Fig. 3H,I), indicating the blocking effect of sANK1 on the nuclear entry of PTBP1. These results show that sANK1 impacted the ratio of PKM2/PKM1 by regulating the distribution rather than the protein expression of PTBP1.

### Overexpression of sANK1 enhanced OXPHOS in PCa cells

**Fig. 4:**
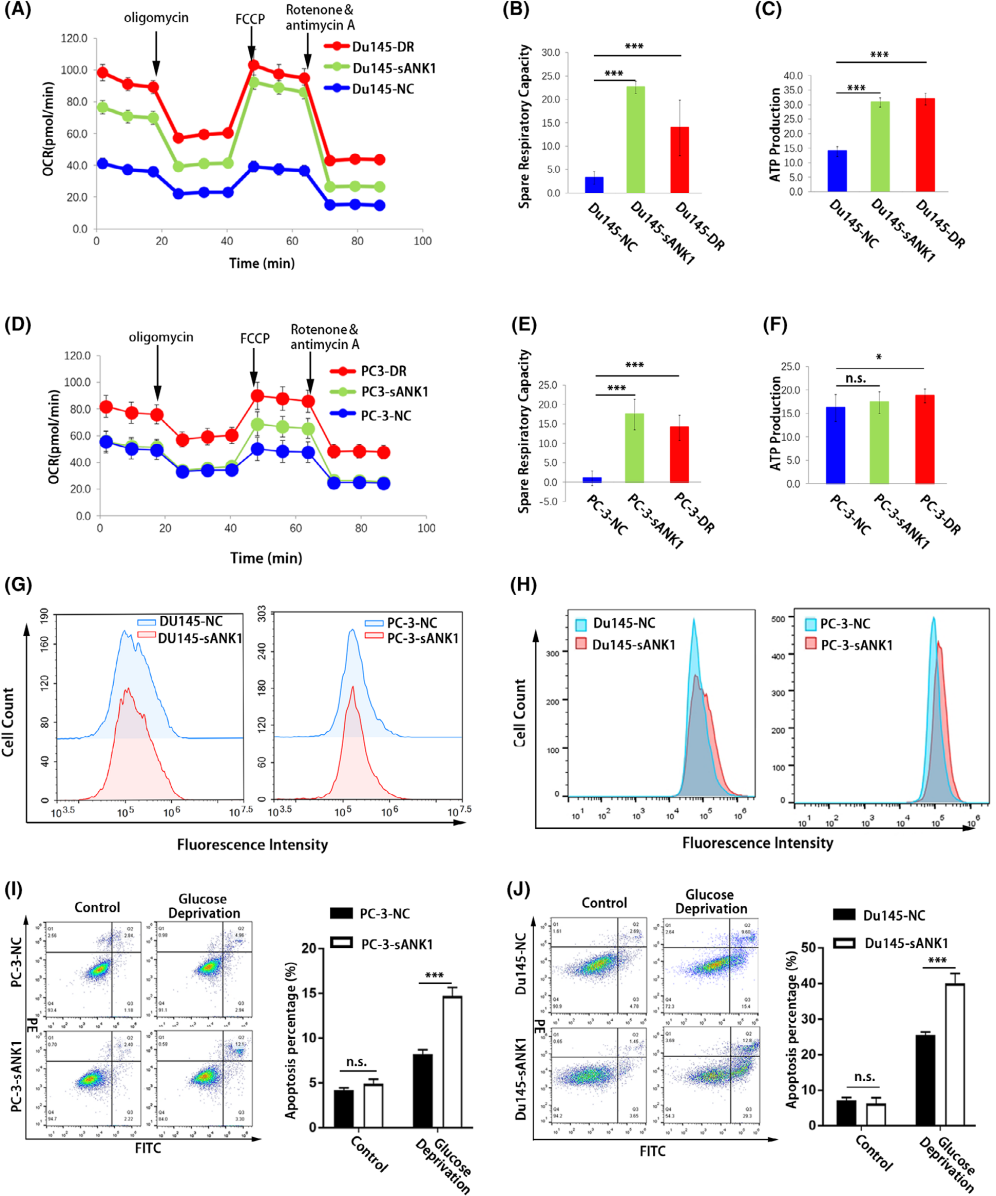
Overexpression of sANK1‐enhanced OXPHOS in prostate cancer. (A) Real‐time OCR measurements in Du145‐NC, Du145‐sANK1, and Du145‐DR cells. OCR values were recorded following sequential treatments with 1.25 μm oligomycin, 2.5 μm FCCP, 0.75 μm rotenone, and 0.75 μm antimycin using a Seahorse XF96 Analyzer. (B) Histogram statistics of spare respiratory capacity in A. The results are representative of three independent experiments. *n* = 8. *P* values, by Student's *t*‐test. ***P < 0.001. Data are mean ± SD. (C) Histogram statistics of ATP production in A. The results are representative of three independent experiments. *n* = 8. *P* values, by Student's *t*‐test. ***P < 0.001. Data are mean ± SD. (D) Real‐time OCR measurements in PC‐3‐NC, PC‐3‐sANK1, and PC‐3‐DR cells. (E) Histogram statistics of spare respiratory capacity in D. The results are representative of three independent experiments. *n* = 8. *P* values, by Student's *t*‐test. ***P < 0.001. Data are mean ± SD. (F) Histogram statistics of ATP production in D. *n* = 8. *P* values, by Student's *t*‐test. n.s., not significant, *P < 0.05. Data are mean ± SD. The results are representative of three independent experiments. (G) sANK1 did not affect the level of intracellular Ca^2+^ in prostate cancer cells. (H) Glucose uptake of PCa cells with or without overexpressing sANK1 was measured using the fluorescence‐labeled glucose analog, 2‐NBDG. The fluorescence intensity was measured by flow cytometry. (I) The apoptosis after cells cultured in glucose‐free medium for 12 h was measured using an Annexin V‐FITC Apoptosis Detection Kit by flow cytometry. Histogram statistics of PC‐3‐NC and PC‐3‐sANK apoptosis percentage. *n* = 3. *P* values, by Student's *t*‐test. n.s., not significant, ***P < 0.001. Data are mean ± SD. The results are representative of three independent experiments. (J) Histogram statistics of Du145‐NC and Du145‐sANK apoptosis percentage. *n* = 3. *P* values, by Student's *t*‐test. n.s., not significant, ***P < 0.001. Data are mean ± SD.

Considering the change in the PKM2/PKM1 ratio, we wondered whether the metabolic pathway shifted when the expression of sANK1 was upregulated. The oxygen consumption rate (OCR) of cells was assessed by using a Seahorse metabolic analyzer in PCa cell lines. The basal respiration was the OCR level before oligomycin treatment, including the oxygen consumption of mitochondrial oxidative phosphorylation and proton leakage. When cells were treated with oligomycin, the decrease in OCR represented the ability to produce ATP. When cells were treated with FCCP, the increase in OCR represented the ability of maximum respiratory capacity and the respiratory potential of mitochondria. Our results showed that both the spare respiratory capacity and ATP production of Du145‐sANK1 and Du145‐DR cells were increased compared with those of Du145‐NC cells (Fig. 4A–C). In PC‐3 cell lines, the spare respiratory capacity and ATP production of PC‐3‐DR cells were higher than PC‐3‐NC cells, but only the spare respiratory capacity was increased in PC‐3‐sANK1 cells while ATP production was unchanged when compared to the PC‐3‐NC cells (Fig. 4D–F). These results revealed that like DTX‐resistant PCa cells, sANK1‐overexpressing PCa cells showed enhanced mitochondrial OXPHOS compared to control cells, suggesting that sANK1 might promote DTX resistance through enhancing mitochondrial OXPHOS of PCa cells. In addition to regulating the ratio of PKM2/PKM1 to enhance mitochondrial OXPHOS, sANK1 was reported to interact with SERCA1 which could affect the level of Ca^2+^ in the myoplasm, and Ca^2+^ is believed to regulate mitochondrial oxidative phosphorylation [22]; we wondered whether sANK1 regulated the OXPHOS via regulating Ca^2+^. As shown in Fig. 4G, we found that the expression of sANK1 did not affect the level of intracellular Ca^2+^ in prostate cancer cells. Furthermore, through treating cells with 2‐NBDG, we found the glucose uptake of Du145‐sANK1 and PC‐3‐sANK1 cells was obviously increased compared to corresponding control cells (Fig. 4H). When cells were cultured in glucose‐free medium for 12 h, a higher apoptosis percentage was also observed both in PC‐3‐sANK1 and Du145‐sANK1 cells than that in corresponding control cells (Fig. 4I,J). These results showed that sANK1‐overexpressing cells were more dependent on glucose metabolism. In summary, the above results demonstrated that overexpressing sANK1 could enhance OXPHOS in DTX‐resistant PCa cells.

## Discussion

Although there have been novel hormonal drugs used in clinical practice, DTX still plays a crucial role in treating advanced PCa [23]. How to address DTX resistance in PCa remains a key challenge for all urologists. In this study, we found that sANK1 was positively associated with DTX resistance in tumor tissues from PCa patients. Further investigation demonstrated that overexpression of sANK1 can significantly induce PCa cell resistance to DTX *in vitro* and *in vivo*. Moreover, we revealed that overexpression of sANK1 could obviously enhance the OXPHOS levels in PCa cells, in accordance with higher OXPHOS levels in the DTX‐resistant PCa cells. We then explored the mechanism underlying sANK1‐mediated DTX resistance and found that sANK1 regulated the ratio of PKM2/PKM1 through PTBP1, thereby enhancing OXPHOS and mediating DTX resistance.

Metabolic reprogramming is an important way for cancer cells to respond to external stress [24]. Cancer cells prefer to use aerobic glycolysis rather than mitochondrial OXPHOS for glucose metabolism and ATP production even in oxygen‐rich conditions, a preference referred to as the famous “Warburg effect” [25]. However, cancer cells can also promote their adaptability and resistance to therapies by adapting their metabolism to different treatments [26]. Reports have shown that increasing OXPHOS levels in tumor cells are an indication of cells resisting chemotherapy [27]. For instance, a recent study described a new cisplatin resistance mechanism in non‐small‐cell lung cancer in which an increased OXPHOS function was the key for the chemotherapy resistance phenotype, proposing the therapeutic exploitability of OXPHOS inhibitors or PGC‐1α downregulation [28]. In addition, a study also revealed that targeting OXPHOS with ALDH inhibitors could reverse drug resistance by blocking autophagy recycling in the mouse xenograft models of various cancers, including PCa [27]. However, the mechanism of metabolic reprogramming in DTX‐resistant PCa cells has rarely been reported. In our current study, we found that overexpression of sANK1 could obviously enhance OXPHOS through PTBP1‐mediated *PKM* alternative splicing, which promotes the resistance of PCa cells to DTX.

PTBP1 is an alternative splicing factor regulating pre‐RNA processing and belongs to the family of heterogeneous nuclear ribonucleoproteins [29]. PTBP1 plays an oncogenic role in many cancers by regulating the alternative splicing of critical genes. In colorectal cancer, PTBP1 is often overexpressed which leads to the alternative splicing of *CD44*, promoting colorectal cancer progression [30]. In glioblastoma, PTBP1 promotes glioblastoma progression by mediating *annexin A7* exon splicing, eliminating its tumor suppressor functions [31]. Only one recent study reported the association between PTBP1 and PCa, which showed that PTBP1 increased in PCa tissues, and its genetic variants affected patient response to androgen‐deprivation therapy in PCa patients [32]. Despite the wide acknowledgment of the alternative splicing function of PTBP1, few studies have focused on the regulation of PTBP1. Here, we found that sANK1, as an integral membrane protein in the endoplasmic reticulum, could interact with PTBP1 and fix PTBP1 to the endoplasmic reticulum membrane, restraining its entry into the nucleus to mediate alternative slicing.

In cancer cells, the most important process involving PTBP1 is glycolysis [29]. Pyruvate kinase (PK) catalyzes the phosphorylation of phosphopyruvate and adenosine diphosphate to produce pyruvate and ATP which is a key rate‐limiting enzyme for glycolysis [33]. The *PKM* gene encodes two subtypes of PK (PKM1 and PKM2), and they show distinctly different regulatory and catalytic features [33]. In some cancer cells, PTBP1 could promote *PKM* splicing to *PKM2* rather than *PKM1*, thus leading to a metabolic shift from OXPHOS to glycolysis [21, 34]. The role of PTBP1 in PKM splicing is already known in several cancer types [21, 34]. In this study, we revealed the relationship between PTBP1‐mediated *PKM* splicing and DTX resistance in PCa. We demonstrated that sANK1‐enhanced OXPHOS promotes the resistance of PCa cells to DTX by suppressing PTBP1‐mediated *PKM* alternative splicing.

## Conclusion

In conclusion, our results revealed that sANK1 was overexpressed in PCa tissues and positively associated with the resistance ability of PCa cells to DTX. Further mechanistic investigation demonstrated that sANK1 could interact with PTBP1 and suppress PTBP1‐mediated *PKM* alternative splicing, thereby decreasing the ratio of PKM2/PKM1, enhancing OXPHOS, and ultimately promoting the resistance of PCa cells to DTX. These findings offer novel potential therapeutic targets to address DTX resistance in PCa.

## Conflict of interest

The authors declare no conflict of interest.

## Author contributions

Most experimental operations and data analysis were conducted by YY and he wrote the article; the language of the manuscript was edited and modified by MD and CJ; HG and WG designed the research. HQ collected the tissue samples and conducted part of the IHC analysis. HY and MC conducted the TMA. WC and WD conducted the paraffin section. All authors read and approved the final manuscript.

## Data Availability

All data needed to evaluate the conclusions are present in the study.
